# Healing beyond the wound: social determinants in chronic vascular care. Perspectives from clinical practice

**DOI:** 10.3389/fsoc.2026.1817248

**Published:** 2026-05-25

**Authors:** Davide Costa, Raffaele Serra

**Affiliations:** 1 Interuniversity Center of Phlebolymphology (CIFL), International Research and Educational Program in Clinical and Experimental Biotechnology, University “Magna Graecia” of Catanzaro, Catanzaro, Italy; 2 Department of Medical and Surgical Sciences, University “Magna Graecia” of Catanzaro, Catanzaro, Italy

**Keywords:** chronic vascular wounds, moral distress, social determinants of health, structural vulnerability, wound care workforce

## Abstract

**Background:**

Chronic vascular wounds are increasingly recognized as complex conditions shaped not only by vascular pathology but also by social determinants of health (SDHs). While research has focused primarily on patient outcomes, limited attention has been paid to how providers experience socially contextualized wound chronicity.

**Objective:**

To explore how wound care professionals understand, experience, and respond to SDHs influencing chronic vascular wound healing, and how these dynamics affect professional identity, moral distress, and workforce sustainability.

**Methods:**

A qualitative study was conducted using semi-structured interviews with 22 wound care professionals (home care nurses and hospital-based specialist nurses) in Calabria, Italy. Participants held post-graduate training in wound care and at least 1 year of clinical experience. Interviews were analyzed using reflexive thematic analysis following Braun and Clarke's six-phase approach.

**Results:**

Five interrelated themes emerged: recognition of upstream social inequalities; structural barriers embedded in clinical practice; invisible advocacy and emotional labor; moral distress linked to constrained professional agency; and implications for professional identity and retention. Providers reframed non-adherence as structurally produced and described chronic wounds as embodied expressions of inequality.

**Conclusion:**

Chronic vascular wounds function as visible markers of social inequality. Addressing healing disparities requires integrating SDHs into clinical frameworks and supporting providers facing structurally generated moral and organizational strain.

## 1 Introduction

### 1.1 Chronic vascular wounds as a growing challenge in wound care

Chronic vascular wounds, including venous leg ulcers, arterial ulcers, and mixed etiology wounds, represent one of the most persistent and resource-intensive conditions in contemporary wound care. Arising from venous or arterial insufficiency, or a combination of both, these wounds are characterized by prolonged healing trajectories, high recurrence rates, and complex management requirements. As populations age and the global burden of chronic disease increases, chronic vascular wounds are increasingly recognized not only as a clinical issue but as a significant public health concern. Epidemiological estimates suggest that chronic leg ulcers affect approximately 1% of the adult population in industrialized countries, with prevalence rising to 3%−5% among individuals over 65 years of age (Star, 2018; Serra et al., 2015). Rates are even higher among very elderly populations and those with multiple comorbidities, and are expected to increase further with the growing prevalence of conditions such as diabetes, cardiovascular disease, obesity, and chronic kidney disease (Sen, 2019).

Clinically, these wounds are associated with persistent exudate, pain, edema, infection risk, and skin changes including lipodermatosclerosis and hyperpigmentation. Healing is often temporary, with recurrence rates for venous leg ulcers reaching up to 30%−50% within 1 year, highlighting that wound closure rarely resolves the underlying vascular pathology (Serra et al., 2015). The economic burden is substantial, driven by repeated clinical visits, advanced therapies, and long-term management, particularly within primary and community care settings (Sen, 2021; Falanga et al., 2022).

Evidence-based management is well established and includes vascular assessment, compression therapy, revascularization where appropriate, infection control, and moisture-balanced wound care (Hinchliffe et al., 2020; Greer et al., 2013). However, outcomes remain highly variable in practice. This variability reflects not only biological and clinical factors but also behavioral, social, and organizational conditions shaping the delivery and sustainability of care (Mitchell, 2025; Avishai et al., 2017).

A critical dimension of this variability lies in the influence of social determinants of health (SDHs). Socioeconomic constraints can limit access to essential resources such as compression garments, adequate housing, transportation, and nutrition, all of which are integral to wound healing. Social isolation and unstable living conditions may further compromise adherence to treatment regimens and continuity of care (Pieper and Di Nardo, 1998; Sen, 2021; Gall et al., 2025; Klein et al., 2021). These factors are not peripheral but central to understanding why standardized interventions may fail in real-world contexts.

Physical limitations and multimorbidity further complicate this landscape. Reduced mobility, often associated with conditions such as osteoarthritis, obesity, stroke, or neuropathy, directly interferes with therapeutic mechanisms such as venous return, undermining the effectiveness of compression therapy (Campbell, 2024). At the same time, coexisting conditions, including diabetes, heart failure, and cognitive impairment, introduce competing clinical priorities and increase vulnerability to delayed healing and complications (Dowling et al., 2025; Costa and Serra, 2025a). As a result, wound care becomes a multidimensional process shaped by the interaction of physiological, functional, and social factors.

Chronicity, therefore, should not be interpreted as a simple failure of clinical management. Rather, it often reflects the limited feasibility of implementing recommended interventions within patients' lived conditions. Treatment regimens such as sustained compression, limb elevation, lifestyle modification, and regular follow-up presuppose stability, resources, and support that may not be available to all patients (Fearns et al., 2017; Falanga et al., 2022).

Healthcare system factors further mediate these dynamics. Fragmented care pathways, time constraints, workforce pressures, and unequal access to specialized services can restrict the delivery of comprehensive and continuous care. Institutional priorities may emphasize measurable outcomes such as healing rates over long-term prevention and social support, thereby reinforcing gaps between clinical guidelines and everyday practice (Frasier et al., 2024; Jain et al., 2025).

Within this context, wound care providers operate at the intersection of biomedical expectations and social realities. They are required to deliver standardized, outcome-driven care while engaging with patients whose capacity to adhere to treatment is shaped by structural constraints. This tension can generate moral and professional strain, particularly when non-healing wounds are framed in terms of individual non-compliance rather than systemic barriers (Sen, 2021). Over time, such conditions may contribute to frustration and burnout, as clinicians navigate responsibilities that extend beyond their formal scope of practice (Frasier et al., 2024; Dhoonmoon, 2024).

Chronic vascular wounds can thus be understood as visible manifestations of broader social inequalities. They often reflect cumulative disadvantage, including limited access to preventive care, chronic disease management, and social resources. In this sense, wound care encounters become sites where structural determinants of health are made clinically visible (Ebbeskog and Ekman, 2001).

At the same time, the longitudinal nature of wound care creates opportunities for more integrated and holistic approaches. Ongoing patient contact may facilitate the identification of social needs and the coordination of interdisciplinary interventions involving clinical and community-based resources (Edwards et al., 2013; Costa and Serra, 2025a). Within this framework, patient education remains relevant but must be context-sensitive, aligning clinical recommendations with patients' functional capacities and everyday realities rather than assuming uniform feasibility (Costa and Serra, 2025a).

Understanding chronic vascular wounds as solely technical problems is therefore insufficient. A more comprehensive approach recognizes them as outcomes of interacting biomedical, social, and organizational processes, requiring models of care that address not only clinical management but also the broader conditions shaping healing trajectories (Costa and Serra, 2026).

### 1.2 The wound care workforce under pressure

The wound care workforce is increasingly described as operating under sustained strain. Across healthcare systems, services dedicated to chronic wound management face staffing shortages, high turnover, and growing workloads, reflecting broader systemic challenges within nursing and community care. Within this context, wound care occupies a particularly demanding position, characterized by cases that are not only clinically complex but also socially and organizationally embedded (Sen, 2021; Lindholm and Searle, 2016).

Recruitment and retention remain persistent challenges. Nursing shortages, burnout, retirement, and migration have reduced the availability of experienced clinicians, while wound care is often perceived as less attractive than acute specialties despite its complexity. In some systems, the lack of formal recognition as a distinct specialty limits career progression and professional visibility (Ennis, 2012; Galazka, 2021). High turnover further exacerbates these pressures, as the loss of experienced staff entails not only technical expertise but also tacit knowledge and relational continuity. Replacing such expertise requires time and resources, creating a cycle in which understaffing contributes to further attrition (Varga and Holloway, 2016).

Chronic wound management presents distinctive demands within this broader workforce instability. Healing trajectories are often prolonged and non-linear, requiring sustained attention within systems increasingly oriented toward efficiency and throughput. Time-intensive care may conflict with productivity expectations, particularly in understaffed settings (Monaro et al., 2021; Fearns et al., 2017; Atkin, 2019). At the same time, the physical demands of wound care, including dressing changes, compression therapy, and patient repositioning, can contribute to fatigue and musculoskeletal strain, especially in community settings where clinicians work with limited support (Lindholm and Searle, 2016; Ogrin et al., 2021).

Emotional demands are equally significant. Chronic wounds are often painful, persistent, and distressing, affecting patients' quality of life and social participation. Clinicians are routinely exposed to these experiences, requiring sustained emotional engagement and regulation. This form of emotional labor becomes particularly challenging when clinical progress is limited or absent (Probst et al., 2025a).

Within organizational contexts, wound care may be both essential and undervalued. Although untreated wounds can lead to severe complications, including hospitalization and amputation, wound services may receive limited resources and visibility compared to other areas of care. At the same time, outcomes such as healing rates are increasingly monitored, often without sufficient consideration of the contextual factors influencing them (Kim et al., 2013; Lindholm and Searle, 2016).

Existing research has largely focused on aggregate workforce indicators such as staffing levels, burnout rates, and job satisfaction. While these metrics are important, they may obscure how specific forms of care work generate distinct forms of strain. Chronic wound care is characterized by its longitudinal nature, its reliance on ongoing patient relationships, and the visibility of non-healing, all of which shape professional experience (Varga and Holloway, 2016).

A key feature is the persistence of non-healing wounds. Unlike other areas of healthcare where improvement may be rapidly observable, chronic wounds often remain unchanged despite adherence to clinical guidelines. This can create a sense of therapeutic stagnation, challenging professional identities centered on healing and improvement (Galazka, 2021; Probst et al., 2025a). At the same time, wounds are visible indicators of bodily deterioration and may be interpreted as signs of treatment failure, generating additional pressure on clinicians.

This dynamic is further complicated by the influence of social determinants of health. Clinicians are trained to deliver evidence-based, holistic care, yet many factors shaping healing, such as financial constraints, limited access to transportation, food insecurity, or cognitive impairment, lie beyond their control. These conditions can undermine treatment plans despite appropriate clinical management (Doughty, 2004; Walburn et al., 2009; Mitchell, 2025).

Awareness of these structural constraints can contribute to moral distress. Clinicians may recognize the most appropriate course of action but be unable to implement it due to resource limitations or organizational barriers. Situations such as insufficient follow-up, restricted access to advanced therapies, or premature discharge due to eligibility criteria create tensions between professional values and practical realities (Deschenes et al., 2020; Zanetoni et al., 2023).

Over time, repeated exposure to these tensions may contribute to burnout, characterized by emotional exhaustion, depersonalization, and reduced professional fulfillment. In wound care, these responses are closely linked to structural conditions, including workload, resource constraints, and limited recognition (Binkanan, 2024; Corbett, 2012; Cubellis, 2018).

Additional pressures arise from generational shifts and skill gaps, as experienced clinicians retire and fewer new professionals enter the field. This transition increases the burden on remaining staff, who must balance clinical responsibilities with mentorship roles, often in the absence of sufficient institutional support (Kim et al., 2013).

Technological and administrative demands further shape the work environment. Documentation requirements and electronic health systems, while intended to enhance accountability, may increase workload and reduce time available for direct patient care. When closely tied to performance evaluation, such systems may be experienced as constraining rather than supportive (Wendland and Taylor, 2017; Clemett, 2025).

Taken together, these dynamics highlight a workforce that is highly skilled yet structurally constrained. Wound care professionals operate at the intersection of clinical complexity and social inequality, often held accountable for outcomes influenced by factors beyond their direct control (Gillespie et al., 2015; Galazka, 2021; Probst et al., 2025a).

Addressing these pressures requires more than workforce expansion alone. It involves recognizing the specific nature of wound care work, including its temporal, relational, and ethical dimensions. Approaches such as interdisciplinary collaboration, structured mentorship, and organizational strategies that contextualize outcomes may help mitigate strain and support more sustainable practice (Hurd et al., 2008).

Ultimately, sustaining the wound care workforce depends on aligning institutional expectations with the realities of care delivery. Supporting clinicians in navigating structural constraints, while acknowledging the limits of individual responsibility, is essential to reducing moral distress and ensuring continuity of care for patients with chronic wounds (Mahmoudi and Gould, 2020).

### 1.3 SDHs and chronic wound healing

The concept of social determinants of health (SDHs) provides a central analytical framework for understanding the unequal distribution, persistence, and recurrence of chronic vascular wounds. SDHs refer to the structural and material conditions in which individuals are born, grow, live, work, and age, including income, education, employment, housing, food security, access to healthcare, and social support (Costa et al., 2023). A substantial body of evidence indicates that these determinants exert a profound influence on long-term health outcomes, often exceeding that of clinical interventions alone. In the context of chronic wound healing, SDHs operate both upstream, shaping the development of vascular disease, and downstream, influencing healing trajectories and their sustainability (Oropallo, 2024; Costa and Serra, 2025a).

Chronic vascular wounds are disproportionately concentrated among populations exposed to socioeconomic disadvantage, multimorbidity, and functional limitations. While these conditions increase the risk of disease onset, SDHs also play a critical role in shaping wound chronicity and recurrence. Healing is not solely a physiological process but one that depends on the feasibility of sustained care practices within patients' everyday environments. When material and social conditions are unstable, even clinically appropriate interventions may fail to produce expected outcomes (Campbell et al., 2024).

Among SDHs, economic resources are particularly influential in mediating access to treatment and continuity of care. Financial constraints can limit the acquisition and consistent use of essential therapeutic materials, while also affecting broader aspects of care such as transportation, nutrition, and caregiving support. As a result, economic disadvantage becomes directly embedded within clinical trajectories, shaping both adherence and long-term outcomes (Cavalcante-Silva et al., 2024).

Housing and living conditions further illustrate the structural dimension of wound care. Clinical recommendations, including hygiene practices, limb elevation, and infection prevention, presuppose stable and adequate environments. When such conditions are not present, the practical implementation of care becomes constrained, highlighting the gap between standardized protocols and lived realities (Kapp et al., 2018).

Nutritional status and food security represent another critical interface between social conditions and biological healing processes. Adequate nutrition supports tissue repair and immune function, while its absence can prolong inflammation and increase vulnerability to complications. Although nutritional considerations are routinely included in clinical guidance, their effectiveness is contingent upon patients' access to sufficient and appropriate resources (Herberger et al., 2020).

Social support also plays a central role in shaping care practices. Chronic wound management often requires sustained engagement with treatment regimens that may be difficult to maintain independently. The presence or absence of supportive networks influences both practical aspects of care and broader psychosocial factors, including motivation, mental health, and adherence (Costa et al., 2023; Ren et al., 2021).

Access to healthcare services, including transportation and geographical availability of specialized care, further conditions healing trajectories. Barriers to access may lead to delays in assessment, interruptions in follow-up, and suboptimal treatment adjustments. Importantly, these structural barriers are often interpreted within clinical settings as individual non-adherence, obscuring their systemic origins (Costa et al., 2024; Jain et al., 2025).

SDHs do not operate in isolation but interact cumulatively across the life course. The convergence of economic, environmental, and social constraints produces compounded vulnerability, within which chronic wounds emerge not as isolated clinical events but as manifestations of broader structural conditions. The concept of structural vulnerability captures this dynamic, emphasizing how health outcomes are embedded within social hierarchies and institutional arrangements (Falanga et al., 2022).

Despite increasing recognition of SDHs in public health, their integration into clinical wound care remains limited. Clinical documentation and assessment frameworks tend to prioritize physiological indicators, while social conditions are inconsistently recorded or addressed. This focus risks narrowing clinical attention to measurable biological parameters, leaving broader determinants insufficiently incorporated into care planning (Sen, 2021).

Performance metrics further reinforce this limitation. Indicators such as healing rates and time to closure are widely used to evaluate care quality, yet they often fail to account for the social contexts in which healing occurs. Without such contextualization, evaluation systems risk attributing socially conditioned outcomes to clinical performance, thereby reproducing structural inequities within healthcare assessment (Cavalcante-Silva et al., 2024).

Integrating SDHs into chronic wound care requires both conceptual and structural shifts. Conceptually, wound healing must be understood as a process shaped by the interaction between biological, social, and material conditions. Structurally, this implies the need for systematic approaches to assessing social needs, integrating contextual information into care planning, and strengthening coordination between healthcare and social support systems. In the absence of such integration, responsibility for addressing social barriers remains localized at the level of individual providers, often without adequate institutional support (Costa and Serra, 2025b).

### 1.4 From patient outcomes to provider experiences

Research on chronic vascular wounds has traditionally focused on patient outcomes, including healing rates, recurrence, cost analysis, and adherence to evidence-based guidelines (Rüttermann et al., 2013). These approaches have generated essential knowledge on key aspects of care such as compression therapy, infection control, and vascular assessment. However, an exclusive emphasis on measurable outcomes provides only a partial understanding of chronic wound care, as it overlooks the organizational, experiential, and contextual dimensions through which care is delivered (Gethin et al., 2020).

Chronic wound management is inherently longitudinal, unfolding over extended periods and often characterized by slow or uncertain progress. The persistence of non-healing wounds introduces a distinct temporal dimension, in which clinicians remain engaged in cycles of assessment and adjustment without clear resolution. This ongoing exposure to clinical uncertainty shapes not only patient trajectories but also provider experience (Eriksson et al., 2022; Ceja Rodriguez et al., 2021).

Outcome-oriented frameworks assume that care quality can be adequately captured through standardized indicators. Yet healing trajectories are influenced by factors that extend beyond clinical technique, including SDHs, institutional constraints, and resource availability. When outcomes are interpreted without reference to these conditions, there is a risk of obscuring the structural context of care and attributing variability primarily to provider performance (Sen, 2024).

Shifting attention toward provider experiences does not diminish the importance of outcomes but situates them within the conditions under which they are produced. Provider well-being, including emotional exhaustion, workload intensity, and organizational support, directly affects continuity of care and patient engagement. In chronic wound care, where sustained relational interaction is central, these dimensions are closely intertwined with clinical outcomes (Probst et al., 2025b).

Although research on healthcare workforce sustainability has documented high levels of burnout, it rarely differentiates between clinical domains (Ielapi et al., 2021; Alshadeedi et al., 2025). Chronic wound care presents specific challenges, combining technical complexity with prolonged exposure to non-resolution and socially mediated barriers to healing (Frasier et al., 2024). Providers must manage physically demanding tasks while also navigating emotionally complex patient interactions, requiring both technical and relational competencies (Von Gunten et al., 2000).

The persistence of non-healing wounds can also challenge professional identity. Clinical training emphasizes measurable improvement, yet in chronic wound care, outcomes often remain uncertain despite adherence to guidelines. This tension may generate a dissonance between professional expectations and practice realities, particularly when responsibility for outcomes is internalized despite awareness of structural constraints (Moffatt et al., 2017).

These dynamics are further shaped by organizational conditions. Time constraints, staffing limitations, and administrative demands can restrict opportunities for comprehensive and relational care. Activities such as patient support, coordination with services, and contextual adaptation, although central to managing chronic wounds influenced by SDHs, are often insufficiently recognized within performance systems (Kim et al., 2013; Davis and Lindell, 2024; Fink-Samnick, 2018).

Chronic wounds can therefore function as focal points of organizational strain. Their management requires sustained allocation of time, resources, and coordination, particularly in constrained settings. Providers are often positioned between patient needs and institutional limitations, negotiating care decisions within conditions of scarcity (Drgac and Himmelsbach, 2023).

Within this context, moral distress may emerge when clinicians are unable to act in accordance with their professional judgment due to systemic constraints. In chronic wound care, this is particularly evident when optimal management would require social or organizational interventions that remain inaccessible. Repeated exposure to such conditions can contribute to emotional fatigue and disengagement (Stechmiller et al., 2019).

Understanding provider experiences therefore expands the analytical lens beyond individual coping to include structural determinants of care work. It highlights how institutional arrangements shape both professional well-being and care sustainability. Incorporating these perspectives allows for a more comprehensive understanding of chronic wound care systems, including the misalignment between biomedical expectations and the social realities of healing (Mahmoudi and Gould, 2020; Lewis et al., 2022).

Such an approach does not replace outcome measurement but contextualizes it. Clinical indicators remain essential, yet their interpretation requires attention to the environments in which care is delivered. Provider experiences offer critical insight into the variability of outcomes and the sustainability of care practices, supporting the development of models that integrate clinical effectiveness with structural awareness (Franks et al., 2016).

### 1.5 Sociological perspectives on care, constraint, and agency

Understanding chronic vascular wound care requires a sociological framework capable of integrating biological processes, institutional structures, and lived experience. While biomedical models explain tissue ischemia, inflammatory dysregulation, and impaired perfusion, they are insufficient to capture the moral, relational, and structural dimensions shaping both healing and professional practice. Chronic wound care unfolds at the intersection of social inequality, institutional constraint, and professional responsibility (Costa and Serra, 2026). To analyze this intersection, the present study draws on complementary perspectives, including the sociology of care work, structural vulnerability, street-level bureaucracy, and the anthropological concept of the social skin (Turner, 2012).

The sociology of care work conceptualizes caring labor as relational, morally charged, and often undervalued (Glenn, 2000). Care extends beyond technical tasks to include sustained engagement with vulnerability and dependency. In chronic wound care, this relational dimension is intensified by the prolonged nature of treatment and the visible deterioration of tissue. Providers accompany patients through uncertainty and marginalization, requiring emotional regulation, interpretive judgment, and moral commitment (Kiaunyte et al., 2021). However, institutional frameworks tend to privilege measurable outputs such as wound reduction and protocol adherence, while less visible activities, including advocacy, coordination, and emotional support, remain underrecognized, creating tension between professional practice and organizational valuation (Macdonald and Merrill, 2002).

The concept of structural vulnerability further situates this tension within broader social hierarchies. It highlights how institutional and economic arrangements systematically constrain health outcomes and life opportunities (Quesada et al., 2011). In chronic wound care, patients often embody cumulative disadvantage linked to poverty, unstable housing, and limited access to care. At the same time, providers operate within resource-constrained systems, where staffing limitations and fragmented infrastructures restrict their capacity to respond. Professional agency is therefore shaped by these structural conditions rather than exercised in isolation (Waters, 2015).

At this intersection, Turner's concept of the social skin offers a critical interpretive lens. The skin functions not only as a biological boundary but as a social surface on which identity and inequality are inscribed (Turner, 2012). Chronic vascular wounds can thus be understood as disruptions of the social skin, where biological damage reflects accumulated social processes. The wound becomes a visible manifestation of structural inequality, rendering otherwise abstract determinants materially present on the body (Costa et al., 2024; Graves et al., 2022).

For providers, engaging with chronic wounds entails repeated encounters with these embodied inscriptions. Clinical interventions such as debridement or compression occur at the interface where social conditions become physiologically visible. This produces a specific form of moral tension: clinicians are tasked with restoring tissue integrity while recognizing that the underlying determinants often remain unchanged (Solomon, 2022).

In this context, professional identity is challenged. Biomedical training emphasizes restoration and measurable improvement, yet chronicity frequently limits the possibility of full healing. As a result, practitioners may redefine success in terms of stabilization, symptom control, or preservation of dignity rather than complete closure (Costa et al., 2024; Ingram, 2014).

Street-level bureaucracy theory further clarifies how such adaptations occur in practice. Frontline providers implement guidelines within conditions of limited resources and competing demands, exercising discretion to align standardized care with patients' lived realities. In chronic wound care, this may involve modifying treatment expectations, prioritizing comfort, or adapting interventions to material constraints. These practices represent forms of constrained agency, situated between institutional requirements and contextual necessity (Hackert et al., 2024).

The interaction between social skin, structural vulnerability, and street-level discretion highlights the layered nature of constraint in chronic wound care. Wounds render inequality visible, institutional conditions delimit possible interventions, and providers respond through negotiated practices that are often informal and insufficiently supported. Agency, in this context, emerges not as full autonomy but as situated and adaptive action within structural limits (Costa et al., 2024).

This sociological synthesis reframes chronic wound care as a domain where biological processes, social hierarchies, and institutional arrangements converge. The wound, understood as social skin, makes inequality visible; care work mediates between embodied vulnerability and systemic constraint; and professional agency is expressed through the ongoing negotiation of what is possible within these conditions (Costa and Serra, 2026).

### 1.6 Theoretical framework

This study is grounded in an interdisciplinary sociological framework integrating the concepts of SDHs (Costa et al., 2023), structural vulnerability, street-level bureaucracy, and the notion of the “social skin” (Turner, 2012).

SDHs provide the macro-level lens through which health inequalities are understood as rooted in socioeconomic and material conditions. Structural vulnerability further extends this perspective by emphasizing how institutional arrangements and social hierarchies systematically constrain both patients' life chances and providers' capacity to deliver care (Costa et al., 2023).

Street-level bureaucracy theory highlights how frontline professionals navigate and adapt policy directives within conditions of limited resources, exercising discretionary judgment in everyday practice (Hackert et al., 2024).

Finally, the concept of the social skin offers a symbolic and material framework for interpreting chronic wounds as visible inscriptions of social inequality on the body (Turner, 2012).

Together, these perspectives inform both data interpretation and the analytical framing of provider experiences.

### 1.7 Study aim and contribution

The aim of this study is to examine how wound care providers understand, experience, and respond to the SDHs shaping chronic vascular wound outcomes, and how these experiences affect their professional roles, moral responsibilities, and workforce sustainability. Rather than focusing on wound healing as a purely clinical outcome, the study centers on the perspectives of home care nurses (HCN) and hospital-based wound care specialist nurses (HBWN) to explore how chronic wounds are encountered as socially conditioned, morally charged, and structurally constrained forms of care work.

Specifically, the study seeks to: (1) analyze how wound care providers interpret the role of social inequality in the development and persistence of chronic vascular wounds; (2) examine how SDHs are translated into everyday clinical obstacles within wound care practice; (3) document the informal, invisible, and emotionally demanding forms of labor that providers undertake to compensate for social and institutional gaps; and (4) explore how these dynamics shape moral distress, professional identity, and intentions to remain in or leave wound care.

The study makes several key contributions to existing literature. First, it extends research on chronic wound care by shifting analytical attention from patient outcomes to provider experiences, highlighting chronic wounds as sites where social inequality is made visible not only on patients' bodies but also in the working lives of care professionals. In doing so, it challenges biomedical and individualizing explanations of non-healing by foregrounding the structural conditions that constrain both patients and providers.

Second, the study contributes to scholarship on SDHs by demonstrating how these determinants are encountered, interpreted, and managed at the point of care. It shows that wound care providers act as frontline interpreters of social inequality, translating upstream determinants into clinical decisions, informal adaptations, and emotional labor, often without institutional recognition or support.

Third, by integrating sociological perspectives on care work, structural vulnerability, moral labor, and street-level bureaucracy, the study provides a theoretically grounded account of why chronic wound care is particularly burdensome for the workforce. It conceptualizes moral distress not as an individual psychological issue but as a structurally produced condition arising from persistent misalignments between professional ideals, institutional expectations, and social realities.

Finally, the study contributes to debates on workforce sustainability in healthcare by illustrating how caring for socially conditioned non-healing wounds shapes burnout, disengagement, and retention decisions among wound care professionals. By situating provider experiences within broader structures of inequality and organizational constraint, the study offers insights relevant to policy, practice, and training, and underscores the need for systemic responses that address both SDHs of wound healing and the conditions under which wound care work is performed.

## 2 Methods

### 2.1 Study design

This qualitative study employed semi-structured interviews to explore how wound care providers understand, experience, and respond to the SDHs influencing chronic vascular wound outcomes. An inductive qualitative approach was selected to capture the complex interactions between clinical knowledge, social context, institutional constraints, and moral responsibility, which cannot be fully addressed through quantitative measures alone.

### 2.2 Participants and settings

Participants included HBN and HBWN involved in managing chronic vascular wounds, including venous leg ulcers, arterial ulcers, and mixed-etiology wounds. All participants had completed a post-graduate diploma in wound care (1-year full-time program following a bachelor's degree) and had a minimum of 1 year of professional experience in chronic wound management. Exclusion criteria included: (1) professionals without direct involvement in the management of chronic vascular wounds; (2) individuals without formal post-graduate training in wound care; and (3) those with less than 1 year of clinical experience in wound care practice. Professionals working exclusively in administrative or non-clinical roles were also excluded. Purposive sampling was used to ensure participants had relevant experience and to capture variation across roles, organizational settings, and levels of seniority. This strategy, widely adopted in qualitative health research, focuses on identifying information-rich cases. Recruitment occurred through professional networks in hospitals and home care settings in Calabria, a southern region of Italy, between 02 January 2020 and 23 December 2025. Recruitment continued until data sufficiency was achieved, that is, when the dataset was considered to provide adequate depth and diversity to support a rich and meaningful thematic analysis. Consistent with the reflexive thematic analysis approach, the aim was not to reach data saturation as a fixed endpoint, but to ensure sufficient information power to develop well-supported and nuanced themes.

### 2.3 Data collection

Following the literature review, an interview guide consisting of open-ended questions was developed and subsequently reviewed by researchers with expertise in qualitative methodologies. Each interview lasted approximately 60–90 min and was conducted by the principal researcher (DC), who had received specific training in interview techniques. Due to organizational or logistical constraints, nine interviews were carried out online. An interview guide (Table 1), developed based on the study's theoretical framework, included open-ended questions exploring:

- Providers' understandings of chronic vascular wounds and healing processes;
- Perceptions of the impact of SDHs, including poverty, housing, nutrition, and social support;
- Experiences of delivering care under time, staffing, and resource constraints;
- Emotional and moral dimensions of caring for non-healing wounds;
- Informal practices, workarounds, and advocacy beyond formal job responsibilities,
- Reflections on professional identity, burnout, and intentions to continue working in wound care.

**Table 1:** Semi-structured interview guide.

Domain	Focus area	Sample questions/prompts	Analytical purpose
Professional background	Role and experience in wound care	Can you describe your role in wound care and how long you have been working in this field?	Contextualize participants' perspectives and level of expertise
Clinical practice	Management of chronic vascular wounds	How do you typically manage patients with chronic vascular wounds? What are the main challenges you encounter?	Explore clinical practices and perceived complexity
Healing trajectories	Experiences of non-healing	Can you describe situations where wounds did not heal as expected? What factors do you think contributed to this?	Examine perceptions of chronicity and clinical uncertainty
Social determinants of health (SDHs)	Impact of social context on healing	In your experience, how do patients' living conditions or social circumstances affect wound healing?	Identify how providers conceptualize SDHs
Structural barriers	System-level constraints	What barriers within the healthcare system affect your ability to provide optimal care?	Explore institutional and organizational constraints
Patient engagement	Adherence and feasibility of care	How do patients manage treatment recommendations such as compression or follow-up visits?	Understand alignment/misalignment between care plans and lived realities
Emotional and moral dimensions	Professional experience and strain	How does working with chronic, non-healing wounds affect you emotionally or professionally?	Investigate moral distress, emotional labor, and burnout
Decision-making and adaptation	Clinical discretion	Do you ever adapt treatment plans based on patients' circumstances? Can you give examples?	Explore street-level discretion and constrained agency
Interdisciplinary and support systems	Collaboration and resources	What kind of support (e.g., social services, team collaboration) is available or lacking?	Assess system integration and resource gaps
Meaning of care	Professional values and definitions of success	What does “successful care” mean to you in chronic wound management?	Examine redefinition of success and professional identity
Closing reflections	Open reflection	Is there anything else you would like to add about your experience in wound care?	Capture emergent themes not covered

All interviews were conducted in Italian, the native language of both participants and the interviewer, to ensure depth of expression and contextual nuance. Interviews were audio-recorded with participants' consent and subsequently transcribed verbatim. No translation was required during data collection or initial analysis. For the purposes of dissemination in English, selected quotations were translated by the research team, with attention to preserving meaning and tone.

### 2.4 Data analysis

Two researchers conducted a reflexive thematic analysis following the approach outlined by Braun and Clarke (2022). The analysis was conceived as an iterative and reflexive process, characterized by active and interpretative engagement with the data rather than a linear, procedure-driven method. The researchers began by repeatedly reading the interview transcripts to develop familiarity with the dataset and to generate initial analytic insights. This phase of immersion was followed by the development of inductive codes through an interpretative process of meaning-making. Coding was therefore understood not as a purely descriptive task, but as an active analytical practice through which features of the data relevant to the research question were identified and interpreted.

Coding proceeded through multiple iterative stages. Initial coding involved close, line-by-line engagement with the transcripts to identify meaningful segments related to participants' experiences, remaining grounded in participants' language. These initial codes were then compared, grouped, and progressively abstracted into broader conceptual categories. Through ongoing processes of comparison and refinement, patterns across interviews were identified and developed into candidate themes. Themes were subsequently refined through iterative discussion between the researchers and repeated engagement with the data. Consistent with a reflexive approach, themes were treated as analytical constructions generated through sustained interpretative work rather than as entities that simply emerged from the data.

Throughout the analysis, sensitizing concepts, including social determinants of health, structural vulnerability, moral labor, and professional agency, informed interpretation while remaining open to revision in response to the data. Contradictions and tensions within participants' accounts were considered analytically meaningful, contributing to the development of a nuanced understanding of the phenomenon under study. In line with reflexive thematic analysis, no claims of triangulation or inter-rater reliability were made, as meaning was understood as co-constructed rather than objectively verifiable.

For example, statements such as “you can't comply with something you can't afford” and “we prescribe what people can't afford” were initially coded as economic barriers to adherence. These were later grouped with codes relating to housing and transport constraints, contributing to the broader theme of structural barriers as everyday clinical obstacles. This illustrates how themes were developed through iterative interpretation of recurring patterns across narratives.

### 2.5 Reflexivity and methodological integrity

Reflexivity was central to the research process and was understood not only as a procedural safeguard but also as an active analytic resource shaping interpretation. The research team—composed of scholars with backgrounds in sociology, medical humanities, and health services research—entered the study with different epistemological sensitivities that influenced how the data were initially perceived and later interpreted.

From the outset, the sociological orientation of some researchers led to a heightened attentiveness to structural determinants of healthcare experiences, including institutional power asymmetries, professional authority, and the role of organizational constraints. This sometimes risked privileging systemic explanations over individual narrative complexity. Conversely, researchers with a medical humanities background were more inclined to foreground experiential, emotional, and narrative dimensions of care, which occasionally led to a stronger empathic reading of participants' accounts and a tendency to interpret distress in relational rather than structural terms. Awareness of these complementary but potentially competing interpretative tendencies became a key part of the reflexive process.

These positionalities were explicitly discussed during team meetings and analytic discussions, where researchers actively questioned how their disciplinary lenses shaped coding decisions and theme development. For example, early interpretations of patient dissatisfaction were repeatedly examined to distinguish whether they reflected participants' accounts or researchers' implicit expectations about healthcare systems. Similarly, there was ongoing critical reflection on the risk of over-pathologising certain narratives of suffering, particularly when interpreting accounts of marginalization in clinical settings.

Analytic memos were used not only to document emerging themes but also to record moments of uncertainty, emotional responses to the data, and instances where prior assumptions appeared to influence interpretation. Rather than attempting to eliminate these influences, the team treated them as part of the analytic process, using them to deepen engagement with the data and to surface alternative interpretations.

Coding and theme development were therefore iterative and grounded in continuous comparison across interviews, but also explicitly informed by reflexive dialogue about how meaning was being constructed. Researcher triangulation did not aim solely at consensus but at productive tension between different interpretative positions, allowing divergent readings to be explored rather than prematurely resolved.

In line with contemporary qualitative reporting guidance, reflexivity was approached as a values-based practice that acknowledges the situated nature of knowledge production (Braun and Clarke, 2025). This strengthened methodological integrity by making visible how researcher positioning shaped, but did not determine, the analytical outcomes.

### 2.6 Ethical approval

The study was approved by the Institutional Review Board of the Interuniversity Center of Phlebolymphology (CIFL) International Research and Educational Program in Clinical and Experimental Biotechnology, approval code: ER.ALL.2018.76.A. All participants received detailed information regarding study aims, procedures, and confidentiality safeguards, and provided written informed consent. Interviews were conducted with attention to emotional safety, and participants could decline to answer any question or withdraw at any time without consequence. Pseudonyms were applied in all transcripts and publications to ensure anonymity. The study was conducted in accordance with the Declaration of Helsinki.

## 3 Results

The study involved 22 health professionals (8 HCN and 14 HBWN). All 22 participants were from Calabria (Southern Italy) and were actively employed as wound care professionals at the time of the study. The sample was pre-dominantly female, reflecting the gender distribution commonly observed in nursing and wound care services. Participants ranged in age from 29 to 62 years, with a mean age of 45.6 years (*SD* = 8.9). Both HCN and HBWN were represented across the age spectrum. All participants held a post-graduate diploma in wound care and had at least 1 year of professional experience in the management of chronic vascular wounds (Table 2).

**Table 2:** Demographic characteristics of participants.

15.6-7,-6.5243ptCharacteristic	*n* (%) or mean (*SD*)
Geographic origin	
Calabria, Italy	22 (100)
Gender	
Women	17 (77.3)
Men	5 (22.7)
Professional role	
Home care nurses	8 (36.4)
Hospital-based wound care specialist nurses	14 (63.6)
Age (years)	
Mean (*SD*)	45.6 (8.9)
Range	29–62
Education and experience	
Post-graduate diploma in wound care	22 (100)
≥1 year of chronic wound care experience	22 (100)

Analysis of interviews revealed a set of interrelated themes that illuminate how wound care providers experience chronic vascular wound care as a form of socially conditioned, morally charged work. Participants consistently framed wounds not merely as clinical problems, but as manifestations of broader social inequalities that shape both patient outcomes and professional lives. Five core themes were identified: (1) recognizing upstream social causes beyond clinical control; (2) structural barriers as everyday clinical obstacles; (3) invisible and informal care work; (4) moral distress, responsibility, and blame; and (5) implications for professional identity and workforce sustainability.

### 3.1 Recognition of social inequalities shaping wound chronicity

Across all care settings, participants demonstrated a high level of awareness of the SDHs shaping chronic wound outcomes.

“After a while you don't even see just the wound anymore, you see the whole life behind it.” (HBWN 2)

“The wound already tells you if someone is poor or alone.” (HCN 3)

Providers routinely linked non-healing wounds to poverty, inadequate housing, food insecurity, limited health literacy, and social isolation.

“These wounds come from poverty before they come from the veins.” (HCN 5)

“It's not one issue, it's everything together.” (HBWN 12)

These factors were described not as abstract risks, but as concrete realities encountered in daily practice.

“You understand it the moment you enter the house.” (HCN7)

“This is not theory for us, it's daily work.” (HBWN 14)

Many participants emphasized that wounds often represented the cumulative effects of wound disadvantage rather than isolated medical events. Providers described patients whose wounds persisted for years despite technically correct care, attributing poor outcomes to living conditions that made healing unrealistic.

As one nurse explained, wounds were often

“The visible end point of a whole life lived with very little support.” (HBWN 10)

This awareness frequently coexisted with frustration. Providers expressed that while they could clearly identify upstream causes, they lacked the means to address them. SDHs were described as “elephants in the room”: widely recognized but rarely acknowledged in formal care planning or documentation. Participants noted that care protocols implicitly assumed stable housing, adequate nutrition, and family support, assumptions that did not reflect the realities of many patients in wound care. Importantly, providers rejected simplistic narratives of patient non-compliance. While they acknowledged difficulties in adherence to compression therapy or lifestyle recommendations, these behaviors were interpreted as responses to constraint rather than as individual failure.

Providers emphasized that “you can't comply with something you can't afford or physically manage,” reframing non-adherence as a structural issue (HBWN 3).

This theme highlights how wound care providers act as informal interpreters of SDHs, translating population-level inequalities into individualized clinical understanding. However, this interpretive labor was largely unsupported by organizational structures, leaving providers to manage the emotional consequences of knowing why wounds fail to heal without being able to intervene meaningfully.

### 3.2 Structural barriers as clinical barriers: when social inequality undermines care plans

Participants consistently described how social conditions functioned as direct barriers to implementing evidence-based wound care. These barriers were not external to clinical practice but deeply embedded within it, transforming social inequality into everyday clinical obstacles.

“Social problems block the treatment before it starts.” (HCN 1) “The guideline assumes a life that doesn't exist.” (HBWN 4)

Housing conditions emerged as a central concern. Providers working in home care frequently encountered overcrowded apartments, lack of basic sanitation, and environments that made hygiene and infection control extremely difficult.

“Housing is one of the biggest enemies of healing.” (HCN 6)

“You can't heal a wound in an unlivable home.” (HBWN 8)

In such contexts, standard recommendations, such as keeping wounds clean and dry or elevating limbs, were often impractical. One provider noted that advising leg elevation was meaningless for patients who slept on sofas or shared beds in small living spaces.

“Elevation is meaningless if the patient sleeps on a sofa.” (HCN 8)

“Protocols don't fit these realities.” (HBWN 5)

Economic constraints were similarly pervasive. Compression stockings, appropriate footwear, and nutritional supplements were often unaffordable, even when partially subsidized. Providers described spending significant time searching for alternative resources, charity support, or improvised solutions, aware that these efforts were often insufficient.

“Compression is a luxury for many patients.” (HBWN 6)

“We prescribe what people can't afford.” (HCN 4)

Transportation barriers further limited access to specialist care. Missed appointments and delayed assessments were common, not due to lack of motivation but because patients lacked reliable means of travel. Providers described the frustration of managing deteriorating wounds while waiting for specialist input that was structurally inaccessible.

“If you don't have a car or money for transport, the specialist might as well not exist.” (HBWN 7)

“It's written as a missed visit, but the real reason is that they simply couldn't get here.” (HBWN 1)

Time and staffing shortages compounded these challenges. Even when providers recognized social barriers, they lacked the time to address them adequately. Wound care visits were described as “rushed negotiations,” in which providers attempted to deliver technical care while simultaneously assessing social needs, educating patients, and providing emotional support.

“You see the social problem immediately, but there's no time to do anything about it.” (HCN 2)

“We recognize the barriers, but the schedule doesn't allow us to intervene properly.” (HBWN 9)

This theme illustrates how structural barriers collapse the distinction between social and clinical domains. Social inequality does not merely influence outcomes; it actively reshapes what counts as feasible care, forcing providers to continuously recalibrate treatment plans and expectations.

### 3.3 Invisible and unrecognized care work: advocacy, improvisation, and emotional labor

A prominent finding was the extent of invisible and informal work undertaken by wound care providers to compensate for social and institutional gaps. Participants described a wide range of activities that fell outside formal job descriptions but were essential to sustaining care.

“We constantly fill gaps that the system leaves open.” (HCN 8)

“If we only did what is formally required, many patients would be abandoned.” (HBWN 11)

Advocacy emerged as a key component of this hidden labor. Providers frequently contacted wound care nurses, family members, and community organizations in attempts to secure basic resources for patients. These activities were described as time-consuming and emotionally draining, yet rarely acknowledged or recorded within organizational systems.

“I'm always calling families, services, anyone who might help.” (HCN 1)

“A lot of time is spent coordinating things that should already be there.” (HBWN 13)

Improvisation was another recurrent practice. Providers adapted treatment plans, modified protocols, and developed creative solutions to accommodate patients' living conditions. While these adaptations reflected professional skill and agency, they were also experienced as precarious, as they often deviated from standardized guidelines without institutional backing.

“You improvise every day, otherwise care just stops.” (HCN 3)

“You adapt the protocol to the person's life, not the other way around.” (HBWN 14)

Emotional labor permeated all aspects of wound care. Providers described managing patients' frustration, shame, and despair, particularly when wounds failed to improve. At the same time, they worked to regulate their own emotions, maintaining professionalism while coping with feelings of helplessness and moral tension.

“The emotional part is constant, not occasional.” (HCN 3)

“Wound care is emotionally heavy work.” (HBWN 14)

Crucially, participants emphasized that this invisible work was taken for granted. Organizational metrics focused on task completion and documentation rather than on relational and emotional aspects of care. As a result, providers felt that a significant portion of their labor remained unseen and undervalued.

“Half of our work is invisible.” (HBWN 5)

This invisibility contributed to feelings of exhaustion and resentment. Providers expressed that they were “doing more and more, but being recognized for less and less,” highlighting the disconnect between actual care work and institutional recognition.

“We're asked to do more every year but valued less.” (HBWN 4)

“There's a huge gap between what we do and what is acknowledged.” (HCN 2)

### 3.4 Moral distress, responsibility, and the burden of blame

Moral distress emerged as a central and pervasive theme. Participants described profound discomfort arising from the gap between what they believed constituted good care and what was possible within existing constraints. Chronic non-healing wounds were a particularly potent source of this distress.

“Moral distress is everywhere in this work, it's part of the job.” (HBWN 10)

“When a wound never heals, the distress just accumulates.” (HCN 5)

Providers frequently internalized responsibility for outcomes, even when they explicitly recognized structural causes. Many described a sense of failure when wounds deteriorated, questioning their competence despite knowing that social factors were decisive. This internalization was reinforced by organizational cultures that emphasized accountability without adequately contextualizing outcomes.

“Even knowing it's structural, you still feel responsible.” (HBWN 7)

“When a wound worsens, you immediately doubt yourself.” (HCN 4)

Blame was described as circulating ambiguously between patients, providers, and systems. While participants rejected blaming patients, they felt that institutional discourses subtly shifted responsibility onto frontline workers. Documentation requirements and audits were experienced as mechanisms that individualized failure while rendering structural constraints invisible.

“Blame is always there, but never clearly placed.” (HBWN 5)

“We don't blame patients, but the institution blames us.” (HCN 6)

The emotional toll of moral distress was significant. Providers reported feelings of frustration, sadness, anger, and, in some cases, emotional numbing. Repeated exposure to non-healing wounds was described as “wearing you down,” leading some to disengage emotionally as a coping strategy.

“Emotionally, this work takes a huge toll.” (HCN 8)

“At first you feel angry and sad, then you feel nothing.” (HBWN 3)

Importantly, moral distress was not framed as an individual psychological weakness but as a collective experience shaped by systemic conditions. Participants emphasized that distress intensified when they felt unsupported or unheard by management, suggesting that organizational recognition plays a critical role in mitigating moral harm.

“This isn't about personal weakness, it's structural.” (HBWN 2)

“What makes it worse is feeling invisible to management.” (HCN 5)

### 3.5 Professional identity and workforce sustainability: “how long can you do this?”

The cumulative impact of structural barriers, invisible labor, and moral distress profoundly shaped participants' professional identities and their views on remaining in wound care. Many providers expressed pride in their work and a strong commitment to patients, yet simultaneously questioned the sustainability of their roles.

“All of these pressures together change how you see yourself as a professional.” (HBWN 1)

“I'm proud of what I do, but I don't know how long I can keep doing it.” (HCN 6)

Chronic wound care was often described as emblematic of broader workforce challenges. Providers felt caught between professional ideals and institutional realities, struggling to reconcile their sense of vocation with the emotional and physical costs of care. Several participants described contemplating leaving wound care, citing chronic exhaustion and lack of recognition.

“You want to do good work, but the system doesn't let you.” (HCN 7)

“Your vocation collides with exhaustion.” (HBWN 8)

Providers reported feeling technically well-prepared for wound care but insufficiently trained to address SDHs, advocacy, and ethical complexity. This lack of preparation intensified feelings of inadequacy and isolation when confronting socially conditioned non-healing wounds.

“Technically I'm prepared, socially I'm not.” (HBWN 11)

“No one teaches you how to deal with poverty and ethics together.” (HCN 3)

At the same time, some participants described developing a resilient professional identity grounded in realism rather than idealism. They reframed success in terms of comfort, dignity, and relationship-building rather than wound closure alone. While this reframing enabled continued engagement, it also reflected an adaptation to constrained conditions rather than a resolution of underlying problems.

“Success becomes making the patient comfortable.” (HCN 4)

“Sometimes dignity matters more than healing.” (HBWN 12)

This theme underscores the link between chronic wound care and workforce sustainability. Providers' decisions to stay or leave wound care were shaped not only by workload but by the moral meaning of their work and the degree to which they felt supported in confronting structural inequality.

## 4 Discussion

The findings of this study suggest the need to reconsider chronic vascular wounds not only as clinical conditions but, according to participants' accounts, as phenomena closely intertwined with social inequality. Nurses consistently described wounds as the visible endpoint of lives characterized by limited support, framing ulceration not merely as tissue breakdown but as the cumulative effect of poverty, unstable housing, food insecurity, reduced mobility, and fragmented healthcare access. While these interpretations derive from participants' perspectives rather than direct measurement of social determinants, they resonate with existing scholarship on SDHs and structural vulnerability, which highlights how disease follows social gradients. In this sense, participants' narratives align with the idea that, in chronic vascular wounds, such gradients may become visible on the skin (Turner, 2012; Costa et al., 2023; Graves et al., 2022).

The nurses' accounts point to what can be interpreted as an epistemological duality in practice. On one level, they rely on biomedical knowledge (e.g., venous hypertension, arterial insufficiency, inflammatory dysregulation, ischemia–reperfusion injury). On another, they described interpreting wounds within patients' living contexts. Observations of housing conditions, food availability, and social support informed their understanding of healing trajectories. This interpretive work was described by participants as a form of “reading” the wound in context. While this study does not objectively assess the diagnostic validity of such interpretations, the accounts suggest that, in practice, wound care may function as a form of informal social assessment alongside clinical evaluation (Weigelt et al., 2022; Costa and Serra, 2026).

Participants also described a perceived mismatch between standardized clinical guidelines and patients' lived realities. Protocols for compression therapy, limb elevation, nutrition, and follow-up were often experienced as difficult to implement under conditions of housing instability, financial constraints, or limited mobility. Importantly, this study captures these challenges as reported experiences rather than as an objective evaluation of guideline applicability. Nevertheless, these perceptions are consistent with literature highlighting barriers to adherence in socially disadvantaged populations (Monaro et al., 2021; Mota et al., 2023).

A key finding concerns how participants reframed non-adherence. Rather than attributing it to individual patient behavior, they frequently interpreted it as structurally constrained. Statements such as “you can't comply with something you can't afford or physically manage” reflect this perspective. While this reframing emerges from participants' viewpoints, it aligns with broader debates in the literature regarding structural vs. individual responsibility in health outcomes. At the same time, it is important to note that this study does not independently verify the relative contribution of structural vs. behavioral factors (Mitchell, 2025; Costa and Serra, 2026).

Participants further highlighted that aspects of patients' social context were often not formally captured in documentation systems. This was described as creating a gap between what clinicians know and what is recorded. However, this should be interpreted as a perception of documentation practices rather than an objective audit of health records. The concept of “epistemic invisibility” is therefore used here as an interpretive lens rather than an empirically measured phenomenon, although it reflects concerns raised in the literature (Mitchell, 2025; Costa and Serra, 2026).

The notion of the “social skin” (Turner, 2012) provides a useful theoretical framework for interpreting these findings. From this perspective, the skin can be understood as a boundary where social conditions may become materially expressed. While this study does not directly test this theory, participants' descriptions of wounds as embodiments of life circumstances are consistent with such an interpretation (Turner, 2012; Costa et al., 2023; Graves et al., 2022).

Participants' accounts also suggest a redefinition of clinical success in some cases. Rather than complete healing, outcomes such as stabilization, comfort, or dignity were described as meaningful. This reframing should be understood as an adaptive response reported by participants, rather than a normative recommendation derived from outcome data. Nonetheless, it echoes discussions in the literature about realistic goal-setting in chronic conditions (Goto et al., 2023).

The study supports interpreting chronic vascular wounds as biopsychosocial phenomena. However, this conclusion is based on qualitative insights into provider perspectives rather than direct measurement of biological, psychological, and social variables. The findings therefore contribute to understanding how clinicians conceptualize wounds, rather than establishing causal relationships (Frasier et al., 2024).

A central contribution of this study lies in showing how participants perceived SDHs not as distant background factors but as immediate barriers within clinical practice. Housing, in particular, was described as shaping the feasibility of care. Reports of overcrowding, dampness, or lack of adequate sleeping arrangements illustrate how recommendations such as limb elevation or infection prevention may become difficult to implement. These observations reflect participants' experiences and are consistent with prior research, although this study does not quantify their impact on outcomes (Pieper and Di Nardo, 1998; Sen, 2021; Gall et al., 2025; Klein et al., 2021; Costa and Serra, 2026).

Economic constraints were similarly described as influencing treatment feasibility. Participants emphasized the cost of compression therapy and related materials. While the study does not include economic data, these perceptions align with literature on financial barriers to adherence (Costa and Serra, 2026).

The study was conducted within the Italian public healthcare system (Servizio Sanitario Nazionale). Participants noted that some elements of wound care (e.g., compression garments, transportation, home support) may involve out-of-pocket costs. These observations are included to contextualize participants' experiences rather than to provide a comprehensive analysis of healthcare financing (France et al., 2005).

Transportation barriers were also described as affecting attendance and continuity of care. Missed appointments were often interpreted by participants as linked to infrastructural limitations. However, this interpretation reflects clinicians' perspectives rather than verified administrative data (Jain et al., 2025).

Time and staffing constraints were reported as limiting the ability to address social needs. Participants described consultations as compressed and requiring prioritization. While these accounts highlight perceived pressures, the study does not include objective workload measurements. Nonetheless, similar challenges have been documented in the literature (Frasier et al., 2024; Dhoonmoon, 2024).

These findings can be interpreted through the lens of structural vulnerability, where both patients and providers operate under constraints shaped by broader systems. This interpretation extends beyond the data and is offered as a theoretical framing consistent with existing scholarship (Olsson et al., 2019).

Participants suggested potential strategies such as integrating social needs screening and improving referral pathways. These should be understood as perceived needs rather than evaluated interventions. However, they align with policy discussions in the literature (Munro and Beck, 2021; Turi et al., 2025).

Another key theme concerns the extent of invisible and informal labor described by participants. Activities such as advocacy, coordination, and emotional support were reported as essential but often undocumented. While this study does not measure the frequency or impact of such activities, their perceived importance was consistently emphasized. This aligns with literature on hidden care work (Galazka, 2021; Drgac and Himmelsbach, 2023).

Improvisation in adapting care plans was also described. These practices reflect participants' efforts to align treatment with patient circumstances. While interpreted here as adaptive expertise, this conclusion remains based on self-reported accounts (George et al., 2024).

Emotional labor emerged as a significant aspect of care. Participants described managing both patient emotions and their own responses. The study does not include psychological assessment, so these findings should be understood as experiential reports, though consistent with existing research (Redmond et al., 2025).

The perceived invisibility of this labor was associated with feelings of misalignment between meaningful work and institutional recognition. Again, this reflects participants' perspectives rather than an objective evaluation of performance systems (Mitchell, 2025; Binkanan, 2024).

The findings also resonate with sociological analyses of care work as gendered and undervalued. This interpretation extends beyond the empirical data but is supported by the predominance of women in the sample and existing literature.

Moral distress was frequently described by participants, particularly in relation to non-healing wounds and perceived constraints. While this study does not measure moral distress quantitatively, the accounts suggest its relevance as an experiential phenomenon. These findings are consistent with prior research (Von Gunten et al., 2000; Moffatt et al., 2017).

Participants described navigating tensions between professional responsibility and structural limitations. Interpretations of blame and accountability emerged as complex and diffuse. These insights are based on subjective reports but align with broader discussions in the literature.

Workforce sustainability emerged as a concern. Participants expressed both commitment and fatigue. While the study does not assess retention outcomes, these perceptions reflect themes identified in health workforce research (Deschenes et al., 2020; Alshadeedi et al., 2025).

Overall, chronic vascular wounds can be understood, based on participants' perspectives, as revealing tensions within healthcare systems, including the interaction between biomedical goals and social realities. While some interpretations presented here extend beyond the direct findings, they are grounded in participants' accounts and supported by existing literature. Future research could build on these insights through mixed-methods or quantitative approaches to further examine the relationships suggested in this study (Frykberg and Banks, 2015).

### 4.1 Study limitations and strengths

This study presents several limitations that should be acknowledged. First, the qualitative design and the context-specific sample limit the transferability of the findings beyond the investigated clinical setting. The research was conducted within a single vascular unit in Southern Italy, a context characterized by specific socio-economic and healthcare system features. As such, the interpretations developed in this study are situated within this particular setting and should be understood in relation to it.

Second, the findings are grounded in participants' accounts and in the interpretative engagement of the researchers with these accounts. In line with reflexive thematic analysis, knowledge is understood as actively constructed through the interaction between participants' narratives, the research context, and the researchers' analytic work. From this perspective, interpretation is not considered a source of bias to be eliminated, but an integral and necessary element of qualitative inquiry that requires ongoing reflexive engagement. The analysis therefore reflects situated meanings co-produced through this process, rather than an objective or detached account.

A further consideration relates to the contextual and temporal specificity of the data. The findings capture participants' experiences at a particular moment within their clinical and social environments and do not aim to represent stable or universal patterns over time.

Despite these limitations, the study has several important strengths. Its qualitative orientation and in-depth analytic approach enabled a nuanced exploration of chronic venous disease beyond a purely biomedical framing, situating wound care within broader social, economic, and institutional contexts. By foregrounding participants' narratives, the study highlights how chronic wounds are understood by clinicians as shaped by the intersection of socio-economic conditions, access to care, health literacy, and healthcare system organization. These interpretations reflect participants' perspectives and are analytically developed rather than empirically measured relationships.

The integration of sociological concepts with clinical perspectives further strengthened the analytical depth of the study, allowing for a multidimensional interpretation that bridges biomedical and social understandings of chronic wound care. These theoretical lenses are used as interpretive tools to make sense of the data, rather than as frameworks empirically tested within the study.

In addition, the study contributes to an underexplored field by focusing on experiences of chronic venous disease in a region characterized by documented healthcare disparities. While the findings are contextually situated, they offer analytically rich insights that may be relevant for reflecting on similar contexts, particularly where comparable social and healthcare conditions exist.

Finally, the reflexive stance adopted throughout the research process, together with transparent reporting of analytic procedures, enhances the credibility and transparency of the study.

Overall, the study does not aim for statistical generalization but instead provides contextually grounded and theoretically informed interpretations, contributing to a deeper understanding of chronic wound care as experienced and interpreted within a specific social and clinical context.

## 5 Conclusion

Chronic vascular wounds are more than persistent lesions of impaired circulation; in participants' accounts, they were described as conditions shaped by multiple social and material constraints. In terms of the study findings, wound care providers perceived these wounds not solely as clinical problems, but as phenomena embedded in broader contexts of poverty, unstable housing, food insecurity, limited mobility, and fragmented access to services. Through these narratives, chronic wounds may be understood as conditions in which the cumulative effects of disadvantage become materially visible. In terms of the literature on embodiment and social inequality, this interpretation is consistent with previous work (Turner, 2012).

In light of the concept of social determinants of health (SDHs), the findings suggest that healing cannot be fully explained through clinical interventions alone. Evidence-based protocols such as compression therapy, vascular assessment, and infection control remain essential; however, according to participants' accounts, their effectiveness appears to be shaped by the material and social conditions in which patients live. As a logical implication beyond the results of this study, when clinical guidelines assume resources that are not available to patients, delayed or non-healing outcomes may be interpreted as structurally conditioned rather than solely as individual or behavioral issues (Donoso, 2021).

In terms of the literature on professional practice, the study highlights how healthcare providers may occupy complex roles at the intersection of clinical care and social vulnerability. Participants' accounts suggest that they often engage in forms of work that extend beyond formal clinical responsibilities, including advocacy, coordination of resources, and emotional support. These aspects of care were described as rarely visible within formal documentation systems and, in participants' views, as contributing to emotional strain and moral distress. Moral distress, as interpreted in this context, may be understood as emerging from the tension between professional values and structural limitations, rather than as an individual-level pathology (Moldovan et al., 2023).

From a broader perspective, and in light of SDHs, chronic vascular wounds may also be interpreted as sentinel conditions that make visible the interaction between social inequality and healthcare delivery. Potentially, they offer insight into how structural disadvantage is encountered and managed within clinical practice. This interpretation extends beyond the empirical findings of this study and should be considered as a conceptual implication rather than a direct result.

These insights suggest that improving chronic wound care may require not only clinical optimisation but also greater integration between healthcare services and social support systems. Interdisciplinary collaboration, improved access to material resources such as dressings and compression devices, and support for transportation and housing instability may represent important components of more equitable care pathways. In terms of implications for training, preparing healthcare professionals to recognize and engage with SDHs and structural vulnerability may support more sustainable and ethically responsive practice (Costa and Serra, 2026).

Overall, this study indicates that chronic wound care, as described by participants, is deeply embedded within social and structural contexts that shape both patient outcomes and professional experiences. While the findings are grounded in participants' accounts, their interpretation suggests that healing cannot be fully separated from the conditions in which it occurs. Potentially, more equitable models of care require not only technical refinement but also greater structural responsiveness within healthcare systems. In conclusion, chronic wound care highlights the intersection between biological processes and social conditions and, in light of the literature, calls for approaches that integrate clinical effectiveness with an awareness of structural determinants of health (Probst et al., 2025b).

## Data Availability

The original contributions presented in the study are included in the article/supplementary material, further inquiries can be directed to the corresponding authors.
